# Multi-exposure speckle imaging through an optical fiber bundle

**DOI:** 10.1117/1.JBO.30.10.106006

**Published:** 2025-10-24

**Authors:** Logan Parker, Shaun A. Englemann, Alankrit Tomar, Andrew K. Dunn, James W. Tunnell

**Affiliations:** The University of Texas at Austin, Department of Biomedical Engineering, Austin, Texas, United States

**Keywords:** speckle, flow, speckle imaging, optical imaging, speckle contrast

## Abstract

**Significance:**

Multi-exposure speckle imaging (MESI) is a label-free technique to visualize and measure blood flow. Accurate perfusion measurements are useful in a variety of applications, including surgery, monitoring treatment, and diagnosing various conditions.

**Aim:**

We aim to demonstrate the feasibility of capturing speckle images through an optical fiber bundle for use in MESI for potential applications such as endoscopy or where free space measurements are not feasible.

**Approach:**

To compare the accuracy of fiber bundle MESI measurements against free space MESI measurements, measurements of a tissue-simulating flow phantom and *in vivo* mouse cortex were acquired simultaneously through free space and an optical fiber bundle.

**Results:**

Using the Pearson correlation coefficient for comparing measurements, $R^{2}$ values of 0.9994 and 0.9942 were calculated for low (1 to 10  μL/min) and high (10 to 100  μL/min) flow rates, respectively. For *in vivo* measurements, an $R^{2}$ value of 0.970 was calculated for flow in 14 vessels and 5 parenchyma regions. $R^{2}$ values of 0.953 and 0.906 were calculated for two vessels before, during, and after a stroke.

**Conclusions:**

MESI measurements through an optical fiber bundle show similar results to free-space MESI.

## 1 Introduction

Blood flow monitoring is critical in a variety of medical applications, including surgery, monitoring treatment, and diagnosing various conditions.^1^^–^^9^ Methods for assessing blood flow include Doppler ultrasound, laser Doppler flowmetry, diffuse correlation spectroscopy, and laser speckle contrast imaging (LSCI). LSCI is a method of visualizing and measuring blood flow without the need for contrast agents.^10^^–^^12^ LSCI utilizes a camera, coherent laser source, and imaging optics to monitor flow in a continuous, wide-field manner.^10^ The sample is illuminated with a coherent laser source, and the reflected speckle pattern is imaged. Areas of flow will induce temporal fluctuations of the speckles, resulting in a blurring effect. By analyzing the speckle pattern, the flow of the sample can be mapped.^10^^,^^12^^,^^13^ Traditional LSCI geometry is through free space, but applications through a fiber bundle have been studied.^14^^–^^18^ Although LSCI can reliably detect changes in flow, it is a relative measure within the sample and experiment. LSCI measurements are also susceptible to artifacts due to changes in optical properties of different tissue types, are highly dependent on the instrumentation used, and do not account for noise.^19^ This is also evident in applications of LSCI using a fiber bundle where changes in flow are detected but absolute values of flow differ between free space measurements and fiber bundle measurements.^16^

To overcome these limitations, multi-exposure speckle imaging (MESI) was developed as the next iteration of LSCI.^19^ MESI captures multiple speckle pattern images at different exposure times. This allows MESI measurements to account for various factors in flow measurements, such as the ratio of dynamic versus static scattering, instrumentation effects on speckle dynamics, and noise.^19^ MESI has been used to measure blood flow more accurately than LSCI in a variety of settings such as cerebral blood flow monitoring and skin microvascular perfusion.^20^^,^^21^ Parthasarathy et al.^22^ demonstrated that MESI can account for static scattering and noise in cortical imaging of thin-skulled mice compared with LSCI. To our knowledge, MESI using a fiber bundle has not been explored in depth.

In multiple endoscopic applications, it is important to assess tissue perfusion for accurate diagnosis of disease or for adequate treatment of a condition. Indocyanine green (ICG) angiography can be used to assess perfusion in different endoscopic procedures, such as assessing anastomotic complications and aneurysm clippings.^23^^–^^25^ ICG angiography requires the use of an external contrast agent to visualize flow, whereas MESI does not. Blood flow measurements from MESI could be added to other endoscopic techniques such as polarization imaging, fluorescence lifetime imaging, and hyperspectral imaging to measure tissue structure, chemical composition, and blood oxygenation.^26^^,^^27^

The results of this paper demonstrate that MESI through an optical fiber bundle provides accurate and similar measurements of flow as traditional free space MESI. A key finding is that the dead space between individual fibers affects speckle contrasts, resulting in skewed values. Instead, each fiber can be segmented from the dead space, and speckle contrast is calculated using intensities from a set number of nearest fibers. By treating each fiber as a sample and calculating contrast using the nearest fiber approach, the effects of dead space are removed from the speckle contrast calculations. Varying flow rates were measured within a microfluidic phantom, and the cortical blood flow in mice was measured* in vivo*. This method of imaging relies on the same principles as free space MESI but can leverage an imaging fiber bundle for potential use in endoscopic applications.

## 2 Materials and Methods

### 2.1 System Design

A 785 nm 90 mW laser diode (L785P090, Thorlabs, Newton, New Jersey, United States) was used to illuminate the sample. Images were acquired at 15 different exposure times ranging from 50  μs to 80 ms. The total time across exposures was 223 ms and with the readout time, one MESI sequence took ∼300  ms to acquire. Kazmi et al.^28^ investigated the tradeoff between acquisition speed and sampling to determine the range of exposure times reliably assessed perfusion *in vivo*. To ensure images at different exposure times had similar levels of pixel intensity, an acousto-optic modulator (AOM, AOMO 3100-125, Gooch & Housego, Ilminster, United Kingdom) was placed in the excitation beam path to deflect varying amounts of excitation light. An iris was placed after the AOM to block higher orders of diffracted light and only allow the first-order diffracted light to reach the sample. A series of mirrors was used to direct the excitation beam to the sample. Two cameras (acA2040-120um, Basler, Germany) were used to image the speckle pattern with a resolution of 2048×1536 of 3.45×3.45  μm sized pixels. These cameras allow for a trigger signal to set the exposure time and start the acquisition of a frame through the GPIO port. To control the AOM and triggering of the cameras, each camera was connected to a data acquisition board (DAQ, USB-6363, National Instruments, Austin, Texas, United States). The DAQ modulated the AOM and triggered each camera frame. To ensure both cameras were simultaneously imaging, they shared a trigger signal from the DAQ. Each MESI sequence started by capturing an image at the shortest exposure time, then capturing an image at the next shortest exposure time, and so on until all 15 images were acquired.

**Fig. 1:**
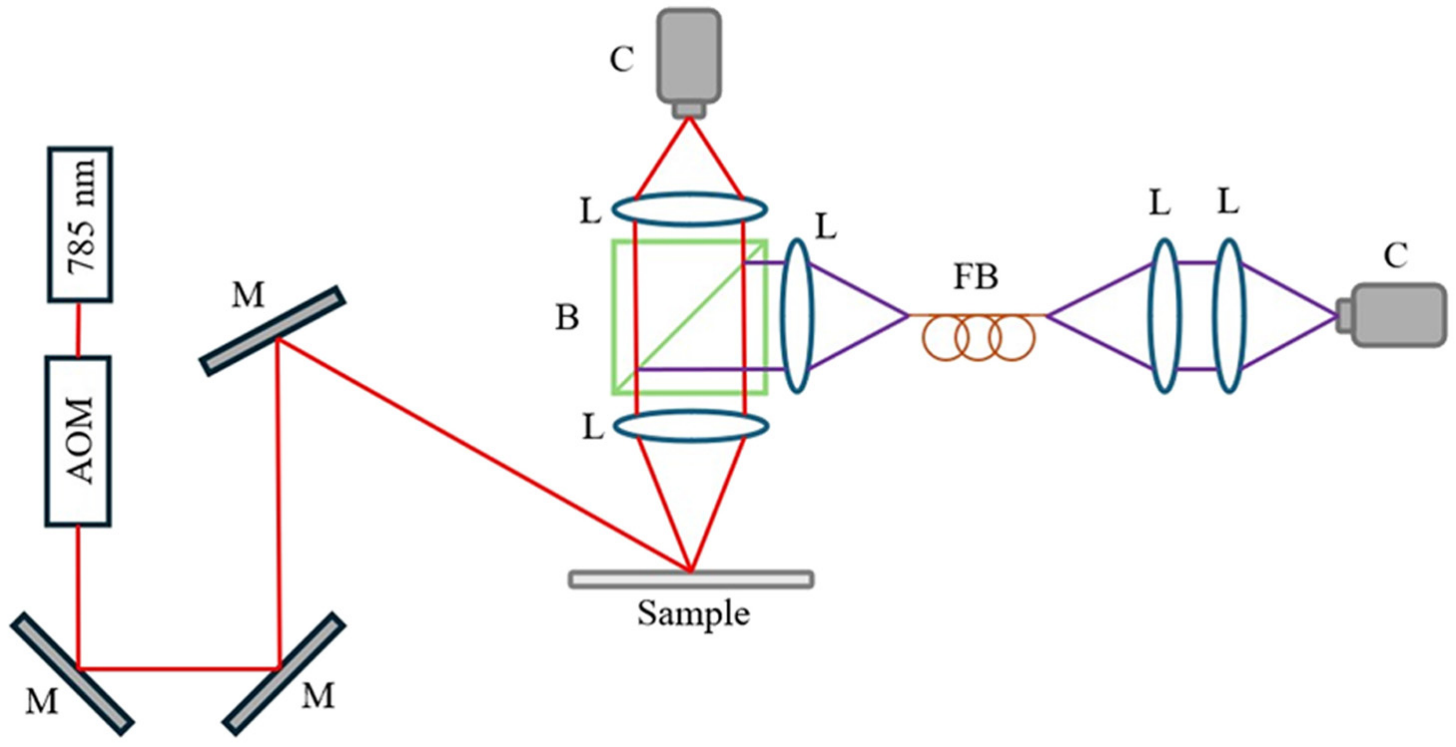
System schematic to simultaneously capture speckle images through the free space path and fiber bundle path. M, mirror; L, lens; B, beam splitter; C, camera; FB, fiber bundle.

The imaging system was constructed to simultaneously capture speckle images through a fiber bundle geometry and a free space geometry (Fig. 1). The fiber bundle has ∼30% transmission at 785 nm, so a 90/10 beam splitter (BSX11R, Thorlabs) created two imaging paths with similar amounts of light at the camera sensor for a given area. The free space geometry consisted of two convex lenses (AF-S NIKKOR 50 mm f/1.8 G, Nikon, Japan) with equal focal lengths to create a 1× magnification of the sample. The speckle size for the free space geometry ranged from 1 to 2 pixels in size. The fiber bundle path consisted of four convex lenses (AF-S DX NIKKOR 55–300 mm f/4.5 to 5.6 G ED and AF-S NIKKOR 50 mm f/1.8 G, Nikon, Japan) and a fiber bundle (Small Diameter Image Guide, Schott, Germany) containing 18,000 individual 7.8  μm fibers. The first pair of lenses had equal focal lengths to image the sample onto the distal end of the fiber bundle with a 1× magnification, so it is on the same imaging plane as the free space camera. The second pair of lenses had different focal lengths to image the proximal end of the fiber bundle onto the camera sensor with a 4× magnification, so individual fibers could be segmented.

### 2.2 MESI Image Analysis

Speckle contrast was calculated by convolving the raw image with a 7×7 sliding window to produce a speckle contrast image using the definition of speckle contrast^10^^,^^12^

$$K=\frac{\sigma }{\left\langle I\right\rangle },$$ (1)

where K is the speckle contrast, σ is the standard deviation of intensities of samples in the window, and ⟨I⟩ is the average intensity. Correlation time, $\tau_{c}$, was calculated using a Levenberg–Marquardt nonlinear least squares algorithm to fit the contrast values of the 15 exposure times of a pixel or ROI to the MESI Eq. (19)

$$K(T,\tau_{c}{)}^{2}=\beta \rho \frac{e^{-2x}-1+2x}{2x^{2}}+4\beta \rho (1-\rho )\frac{e^{-x}-1+x}{x^{2}}+\nu ,$$ (2)

where T is the exposure time, β is the instrumentation factor, ρ is the fraction of dynamically scattered light, and x is the $T/\tau_{c}$. ICT (inverse correlation time) is $1/\tau_{c}$. Fittings for each experiment were done independently of the results of other experiments. For each experiment, β was first fitted for each flow rate or ROI, and then, the averaged β was determined. The data from that experiment would be fitted again to determine ICT with a constant β. For the microfluidic experiments, 100 MESI sequences were acquired for each flow rate. For both the free space and fiber bundle images, an ROI across the full width of the channel and ∼500  μm along the channel was selected. The average contrast within the ROI was calculated for each frame, and then, the contrast for each exposure time was averaged across the corresponding 100 frames. For the *in vivo* mouse cortical imaging, a similar procedure was conducted for each ROI where 100 MESI sequences were acquired, and average contrast was calculated. For the *in vivo* stroke images, MESI sequences were acquired continuously during stroke induction and for 10 min after clot formation. Average contrast was calculated for ROI’s in each frame, but contrast was not averaged across different frames so that a time course of dynamic flow rates and ICT values were acquired.

### 2.3 Microfluidic Experiments

As a first step for discriminating among different flow rates, a microfluidic flow phantom exhibiting laminar flow was used to capture MESI measurements for 1 to 100  μL/min flow rates. For microfluidic experiments, a solution of 1.1  μm polystyrene microbeads (5100A, 10%w/w, Thermo Fisher Scientific, Waltham, Massachusetts, United States) was diluted with DI water in a 4.8% v/v to achieve similar scattering properties to blood ($\mu_{s}=25\;{\mathrm{mm}}^{-1}$, g=0.92).^29^^–^^32^ The microfluidic phantom contained a 300  μm square cross section channel inside a medium of Titanium dioxide (CAS 1317-80-2, Sigma, Kawasaki, Japan) and polydimethylsiloxane (Sylgard 184 P DMS, Dow Corning, Midland, Michigan, United States). The PDMS was cured in a 10-to-1 ratio with a curing agent, and ${\mathrm{TiO}}_{2}$ was added (1/8% w/w) to mimic the scattering properties of tissue $\left(\mu_{s}^{\prime }∼8\;{\mathrm{cm}}^{-1}\right)$.^30^ Flow of the microbead solution was controlled using a syringe pump (ALADDIN-1000, World Precision Instruments, Sarasota, Florida, United States) connected to the phantom’s channel. Two sets of flow rates were captured to simulate a wide range of flow speeds. Low rates of 1 to 10  μL/min in 1  μL/min increments are equivalent to flow speeds of 0.2 to 2  mm/s for the channel size and are similar to flow speeds of capillaries in the mouse parenchyma.^33^^–^^35^ High rates of 10 to 100  μL/min in 10  μL/min increments are equivalent to flow speeds of 2 to 20  mm/s for the channel size and are similar to flow speeds of vessels in the mouse cortex.^35^ ICT was calculated through both imaging paths and compared utilizing the Pearson correlation coefficient (PCC) to determine similarities between the free space and fiber bundle measurements.

### 2.4 *In Vivo* Mouse Experiments

*In vivo* imaging of the mouse cortex was performed through chronic cranial windows.^36^ The mouse was anesthetized during all imaging sessions and stroke procedures using isofluorane.^8^^,^^30^ All animal procedures were approved by the Institutional Animal Care and Use Committee (IACUC) of The University of Texas at Austin. Multiple areas of the cortex within the cranial window were imaged to select a variety of vessels and parenchyma regions. For each ROI, average ICT was calculated through each imaging path and compared utilizing PCC to determine similarities between the free space and fiber bundle measurements.

Photothrombotic stroke was induced through retro-orbital injection of rose bengal dye (15  mg/kg, Sigma) and illumination of 20 mW 532 nm laser light onto a ∼250  μm diameter point of the mouse cortex.^8^^,^^37^ A live feed of the 80 ms speckle contrast images from each MESI sequence was used to visually determine when flow decreased or increased. The 532 nm laser light was left on until the speckle contrast of the target vessel matched the speckle contrast of the surrounding parenchyma region. During and after stroke induction, images of downstream vessels were captured. Average ICT values of vessel and parenchyma ROI’s were compared utilizing PCC to determine similarities between the results of the two imaging paths.

### 2.5 Fiber Bundle Contrast Calculation

**Fig. 2:**
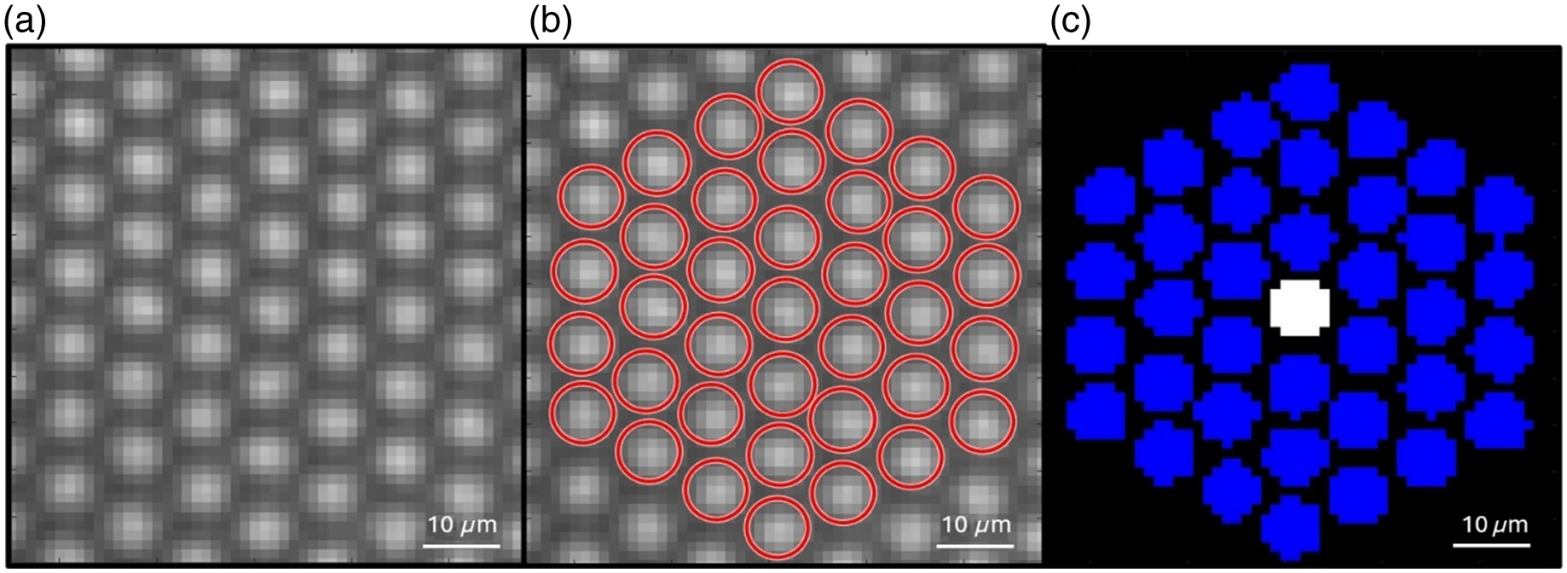
Example image of the proximal end of a fiber bundle under even illumination for segmenting individual fibers for contrast calculations. (a) Proximal end of the fiber at 4× magnification. (b) Segmentation of individual fibers. (c) Pixels corresponding to nearest fibers used in contrast calculation (blue) for the fiber of interest (white).

To calculate speckle contrast through a fiber bundle, a different approach to the traditional sliding window was used. Each fiber was treated as a sample point, and a method of calculating contrast with the nearest fibers was used. A magnification of 4× was used to ensure that one pixel was not imaging a single fiber and an undetermined amount of dead space. Before each experiment, a fiber mask was created by capturing images at 100 ms exposure time and adjusting the laser intensity so as not to oversaturate the image. NIR light was used to evenly illuminate the fiber bundle for segmentation [Fig. 2(a)]. Segmentation was then performed using MATLAB’s imfindcircles command, and each fiber was assigned a list of corresponding pixels [Fig. 2(b)]. This mask was then applied to the raw imaging data. For each image frame, the average pixel intensity was calculated for each fiber. Then, using the nearest 36 fibers, contrast was calculated as a standard deviation over the mean of the average pixel intensity calculated for each fiber intensity [Fig. 2(c)]. Free space speckle contrast is calculated using a 5×5 or 7×7 window, which is 25 or 49 pixels, respectively. Three rings around any fiber resulted in a number between 25 and 49, whereas two or four rings would be outside of that range.

## 3 Results

### 3.1 Fiber Bundle Speckle Contrast Measurements

**Fig. 3:**
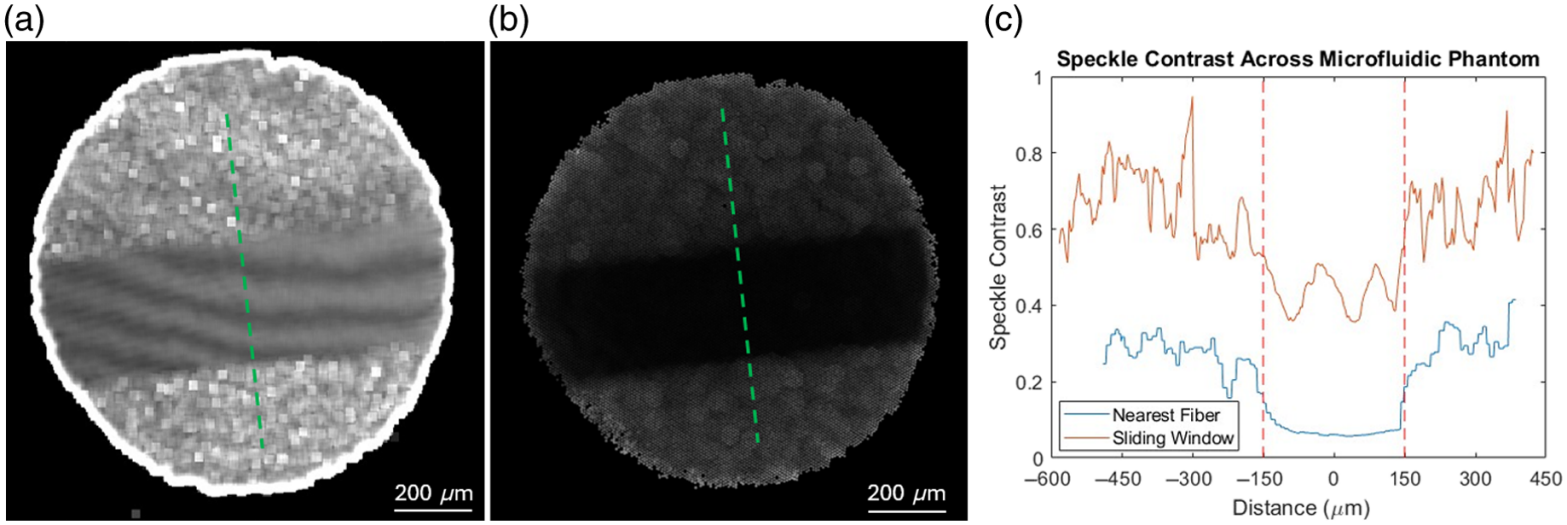
Comparison of contrast calculation between the sliding window and nearest fibers methods. Images captured at 10  μL/min and 7.5 ms exposure time. (a) Cropped contrast image for sliding window at 1× magnification. (b) Contrast image for nearest fibers at 4× magnification. (c) Line plot of contrast across each channel [shown in green in panels (a) and (b)]. The edges of the microfluidic are denoted by the dashed red lines.

To determine how to calculate speckle contrast through a fiber bundle, two approaches were taken: sliding window and nearest fiber. For computing speckle contrast through the traditional 7×7 sliding window, magnification of the proximal end of the fiber was 1× [Fig. 3(a)]. This ensured that no individual pixel was only sampling the dead space between the fibers. This geometry resulted in varying contrast values within the channel, despite the presence of laminar flow. Taking the proposed approach of increasing magnification and segmenting individual fibers resulted in more stable contrast values along the channel [Fig. 3(b)]. The contrast-to-noise (CNR) ratio of the free space signal is 3.0, and the CNR of the fiber bundle signal is 5.5. The results of the microfluidic and *in vivo* mouse experiments use the latter approach.

**Fig. 4:**
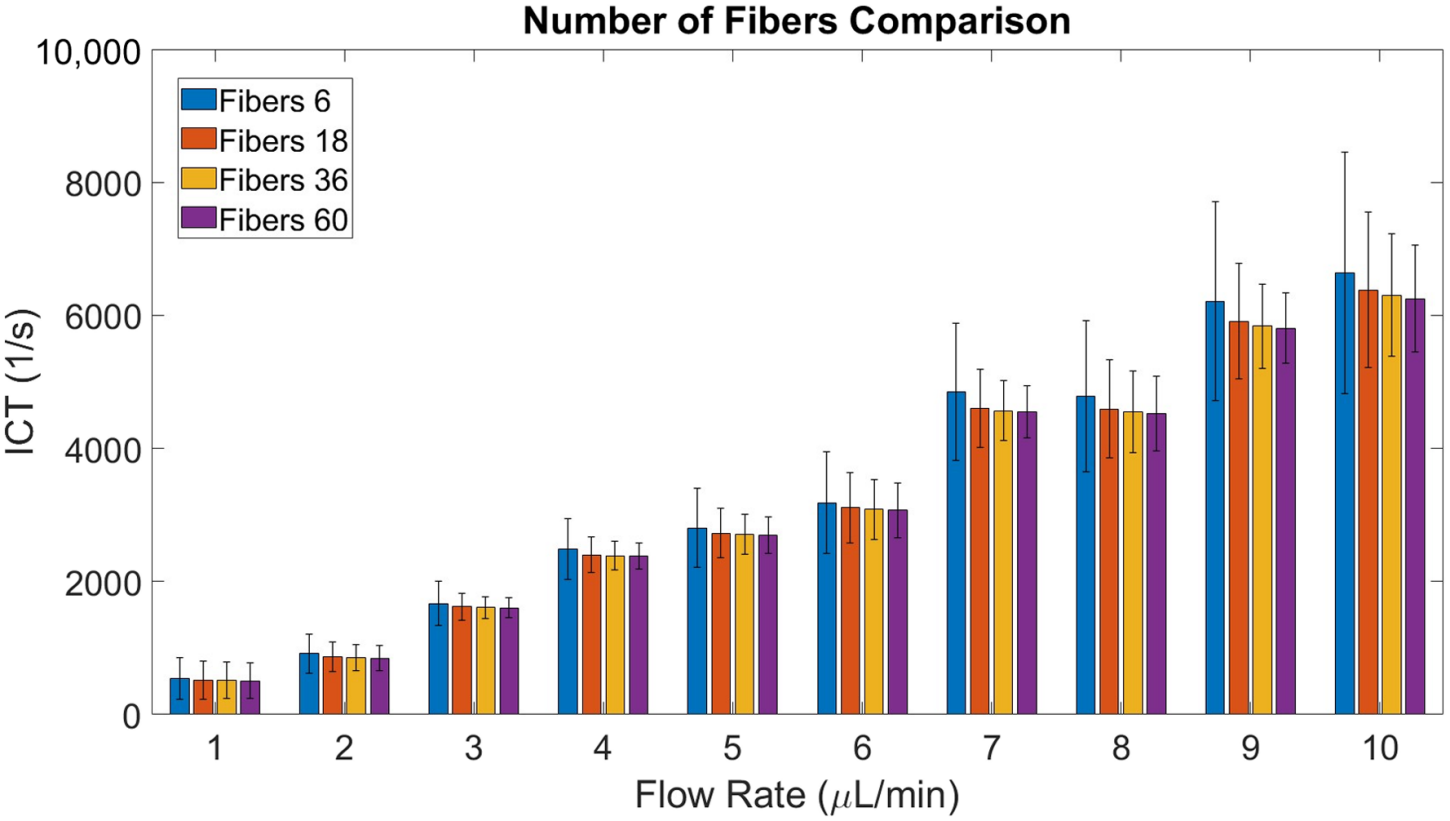
Average ICT value between fibers when calculating speckle contrast using the nearest 6, 18, 36, or 60 fibers; ICT is calculated for each fiber individually. Error bars represent the standard deviation of ICT.

The trade-off between accurate measures of ICT versus spatial resolution was explored. Speckle contrast using the nearest 6, 18, 36, and 60 fibers was explored as these are the number of fibers to form 1, 2, 3, and 4 rings around an individual fiber, respectively. For the microfluidic experiment at low flow rates, an ROI within the middle of the channel was taken. ICT was calculated for each fiber based on how speckle contrast was calculated. The average ICT and the standard deviation of ICT values between fibers were calculated (Fig. 4). To balance the tradeoff between ICT accuracy and spatial resolution, the 36 nearest fibers were used for all subsequent experiments.

### 3.2 Microfluidic Experiments

**Fig. 5:**
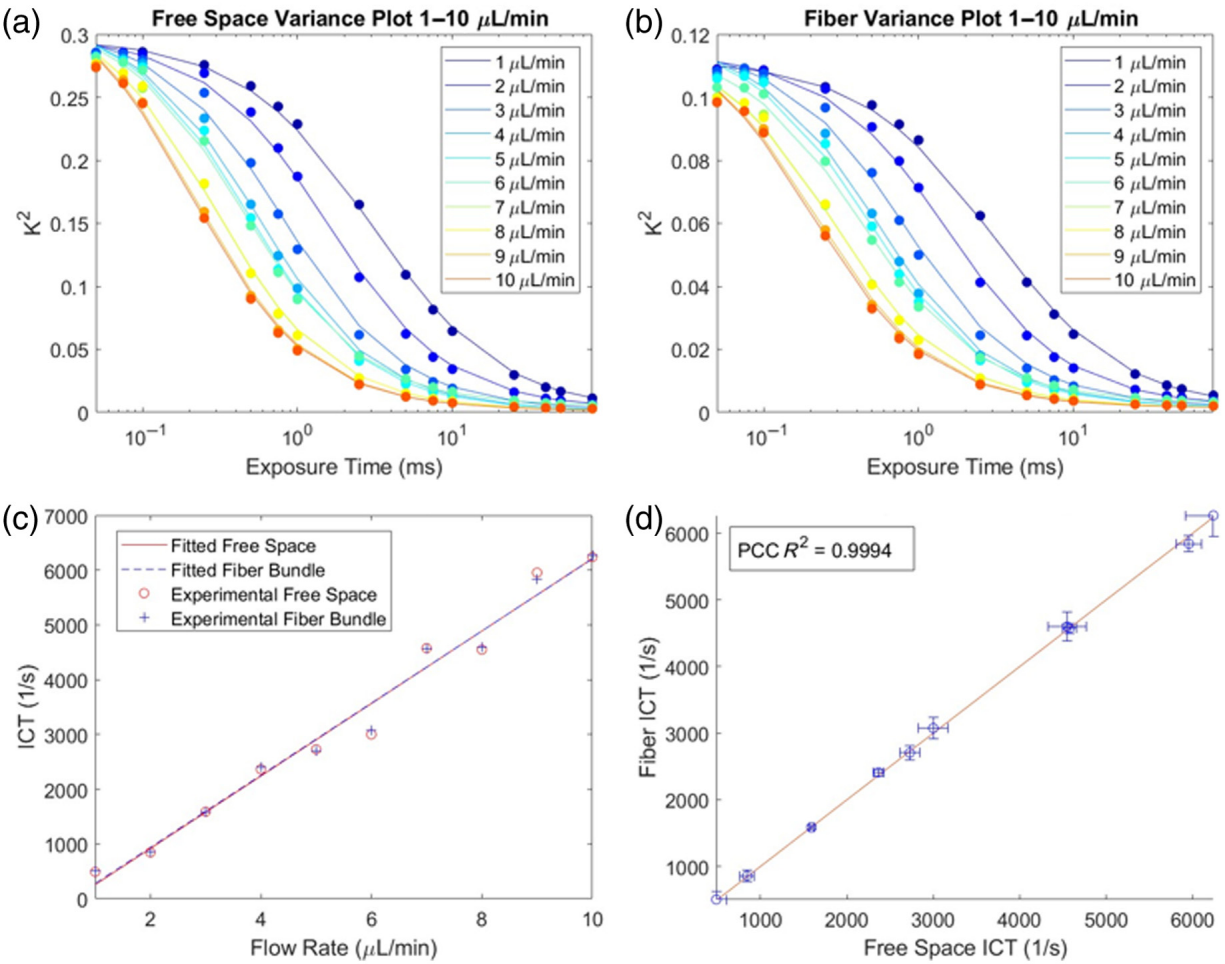
Results of low flow rate measurements from 1 to 10  μL/min. (a) Speckle variance (speckle contrast squared) plot of each flow rate for the free space geometry. Circled points represent experimental data, and the line plots represent the fitted speckle variance data. (b) Speckle variance plot of each flow rate for the fiber bundle geometry. Circled points represent experimental data, and the line plots represent the fitted speckle variance data. (c) ICT plot of flow rate for both the free space geometry and the fiber bundle geometry. (d) ICT comparison plot with standard deviation of ICT values between the fibers, with the ROI shown with bars and a 1:1 fit line.

To determine the predictive value of measured ICT against flow rate, a series of images were captured of a microfluidic flow phantom at a range of flow rates. For each flow rate, 100 MESI sequences were captured, and the ICT value was calculated based on the average speckle contrast values for each set of frames at the same exposure time [Figs. 5(a) and 5(b)]. For the lower flow rates of 1 to 10  μL/min, the linear regression of the free space and fiber bundle results had $R^{2}$ values of 0.978 and 0.981, respectively [Fig. 5(c)]. Comparing the ICT values from the fiber bundle path against the free space ICT results, the $R^{2}$ of the PCC is 0.9994 [Fig. 5(d)].

**Fig. 6:**
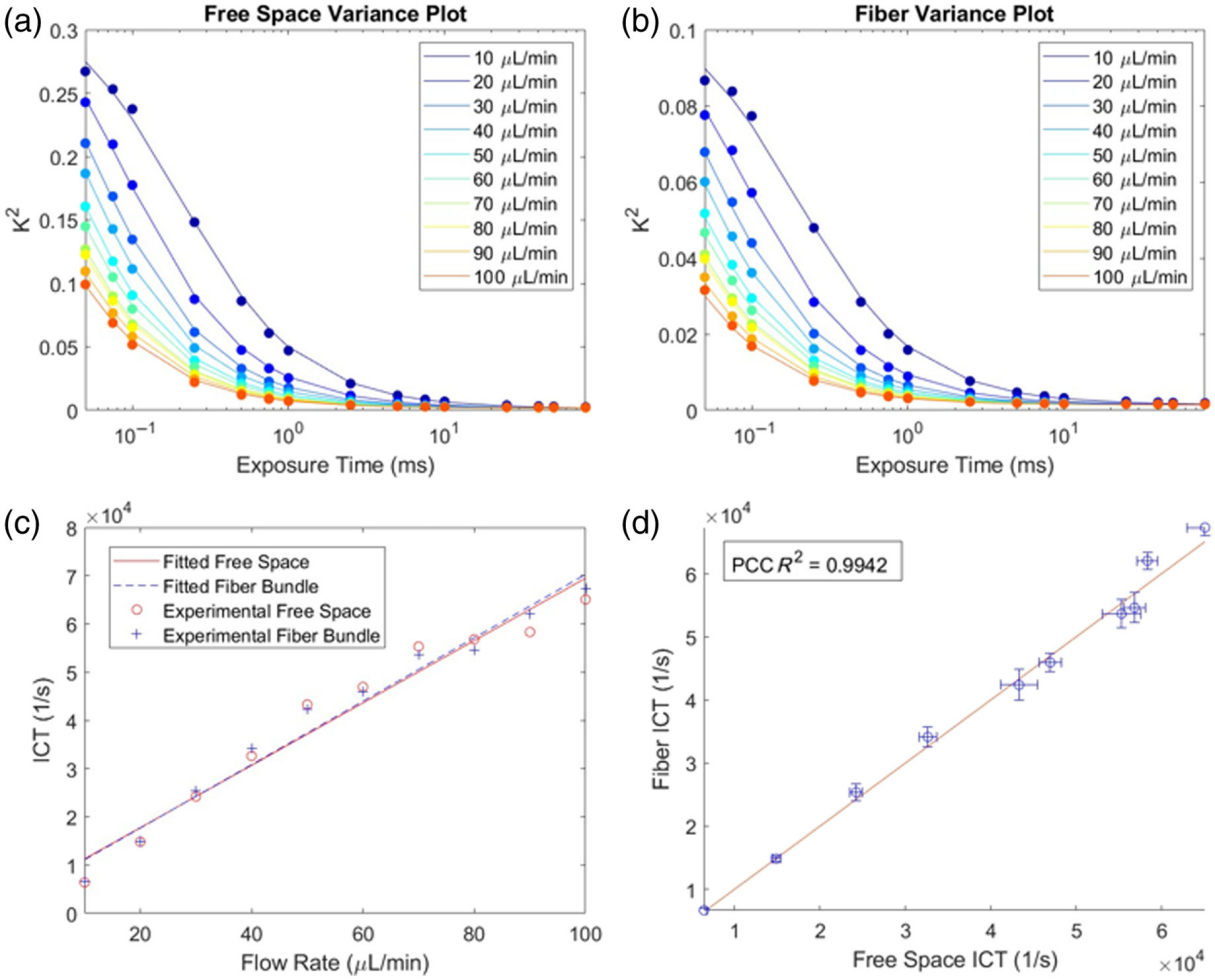
Results of high flow rate measurements from 10 to 100  μL/min. (a) Speckle variance plot of each flow rate for the free space geometry. Circled points represent experimental data, and the line plots represent the fitted speckle variance data. (b) Speckle variance plot of each flow rate for the fiber bundle geometry. Circled points represent experimental data, and the line plots represent the fitted speckle variance data. (c) ICT plot of flow rate for both the free space geometry and the fiber bundle geometry. (d) ICT comparison plot with standard deviation of ICT values between the fibers, with the ROI shown with bars and a 1:1 fit line.

The free space speckle variance is consistently higher than the fiber bundle speckle variance at corresponding flow rates and exposure times [Figs. 6(a) and 6(b)]. For the higher flow rates of 10 to 100  μL/min, the linear regression of the free space and fiber bundle results had $R^{2}$ values of 0.957 and 0.974, respectively [Fig. 6(c)]. Comparing the ICT values from the fiber bundle path against the free space ICT results, the $R^{2}$ of the PCC is 0.9942 [Fig. 6(d)].

### 3.3 *In Vivo* Mouse Experiments

**Fig. 7:**
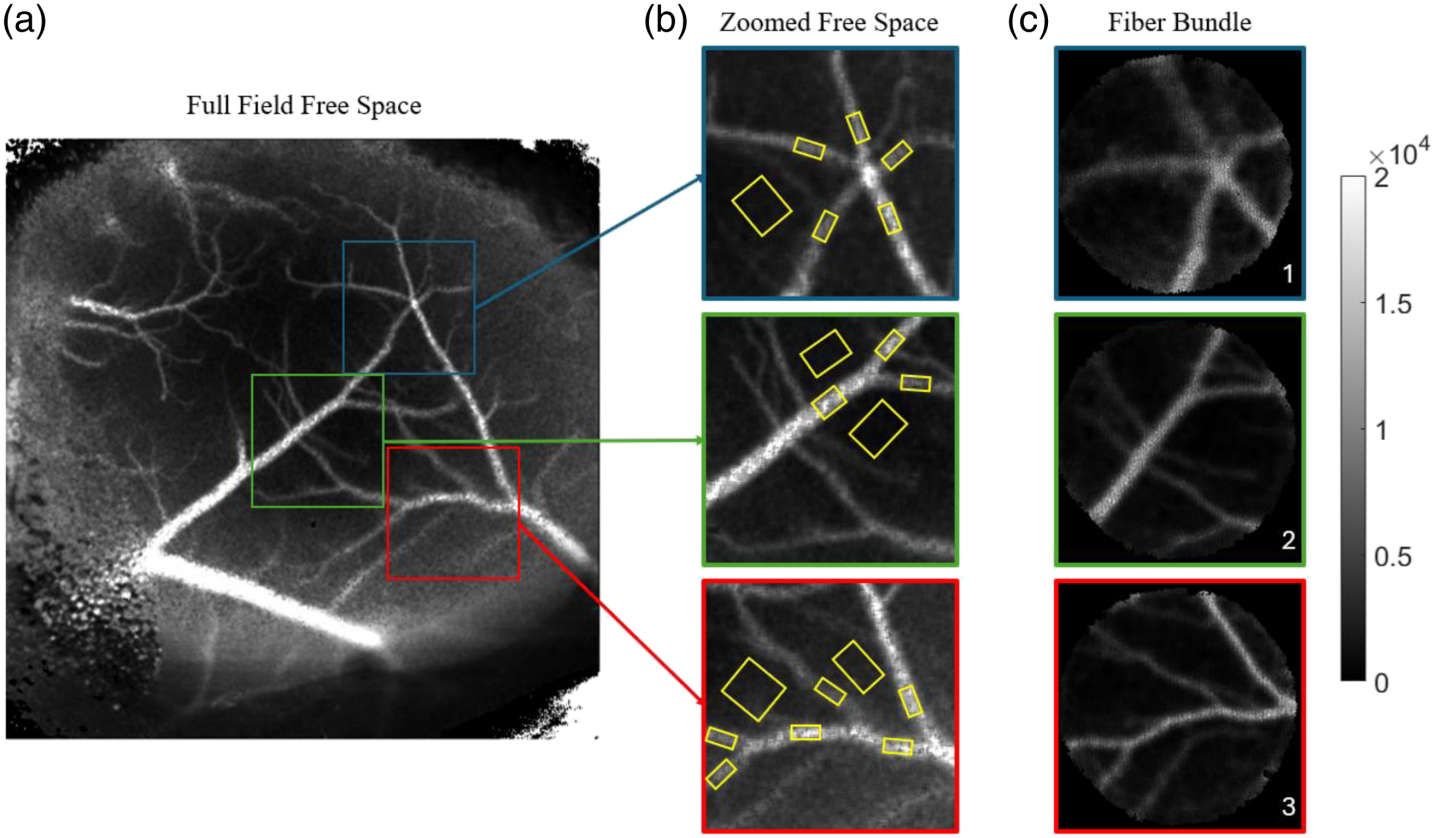
(a) Full-field free-space ICT image of the mouse cortical surface. Three regions were imaged with the fiber bundle shown in panel (c). Corresponding regions from the free space path are shown in panel (b). Included in the free space images are the vessel and parenchyma ROI’s shown in yellow.

**Fig. 8:**
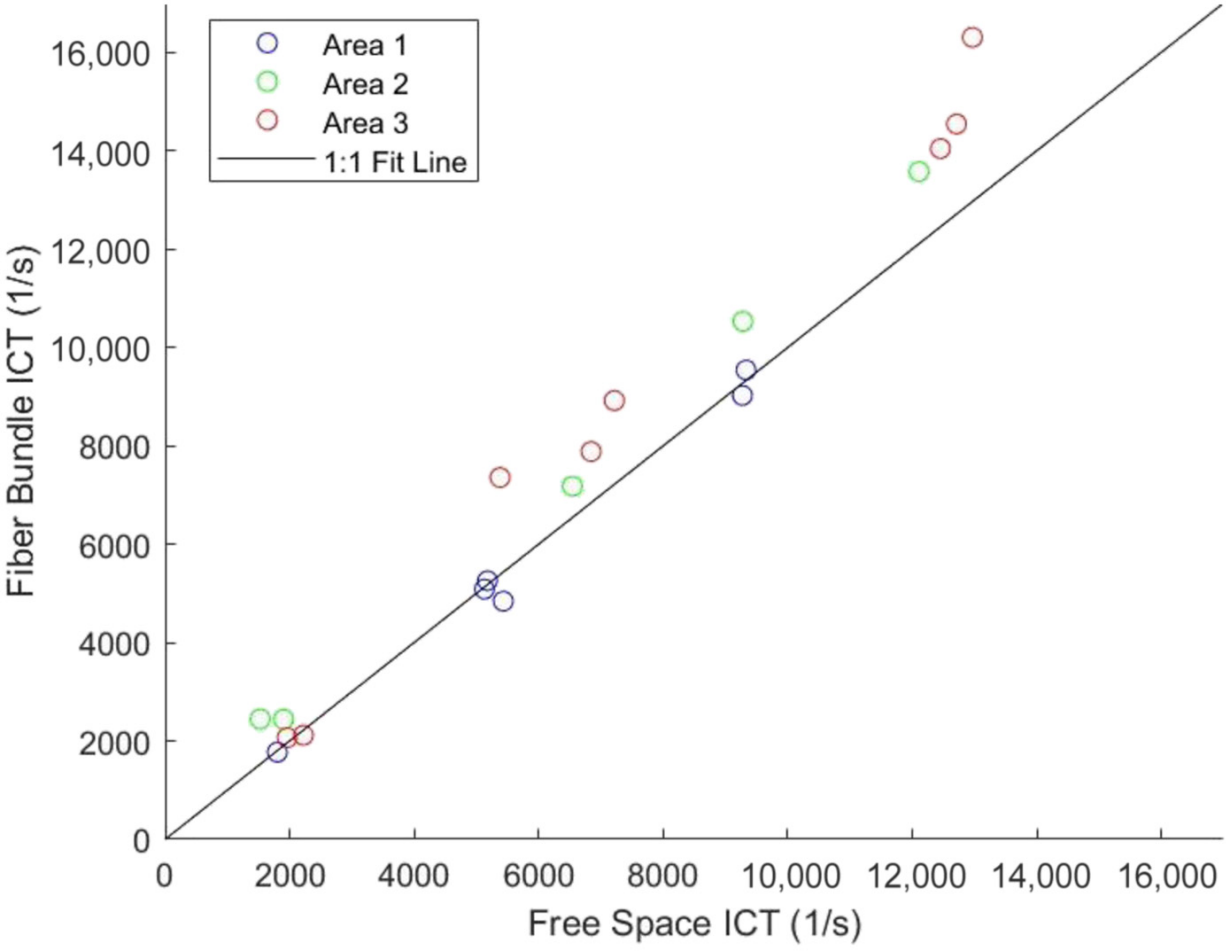
ICT comparison chart between the free space and fiber bundle paths. Each region from Fig. 5 is plotted in corresponding colors with the 1:1 fit line.

To determine the accuracy of *in vivo* ICT measurements, mouse cortical images were captured at baseline and during a stroke. For the mouse under anesthesia experiment, 14 vessels were selected, and five parenchyma regions were selected (Fig. 7). Comparing the ICT values from the free space path and fiber bundle path through PCC, an $R^{2}$ of 0.970 was found (Fig. 8).

**Fig. 9:**
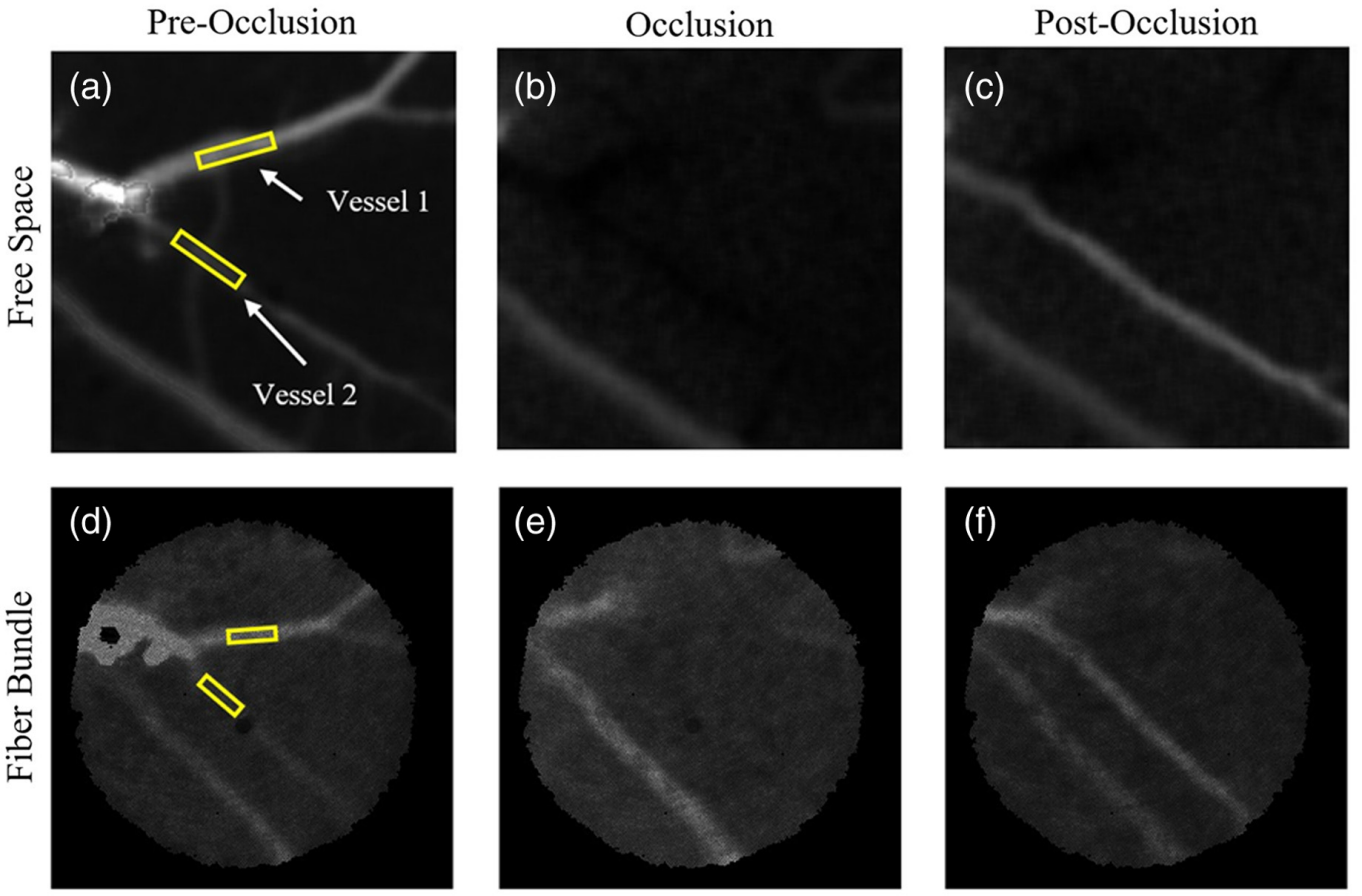
Free-space images are shown on the top row before occlusion (a), during occlusion (b), and after occlusion (c). The fiber bundle images are shown on the bottom before occlusion (d), during occlusion (e), and after occlusion (f). The two vessel ROI’s are shown in the pre-occlusion images with yellow outlines.

For the stroke experiment, two ROIs of different vessels were selected (Fig. 9). The stroke was induced on a vessel that branched into two smaller vessels. Both ROIs were selected downstream of the stroke location to minimize the effects of the green laser light on speckle contrast measurements [Figs. 9(a) and 9(d)]. Once the clot formed upstream, the ICT of both vessels lowered to the background parenchyma [Figs. 9(b) and 9(e)]. Once flow stopped, the laser was turned off, and the stroke induction was halted. Part of the clot cleared, and flow returned to one of the vessels [Figs. 9(c) and 9(f)]. For both vessels, the start of the occlusion is defined as the ICT within the vessel dropping below $1500\;s^{-1}$. For vessel 2, post-clearance was defined as when ICT was above $1500\;s^{-1}$ and stayed above the threshold for at least 60 s.

**Fig. 10:**
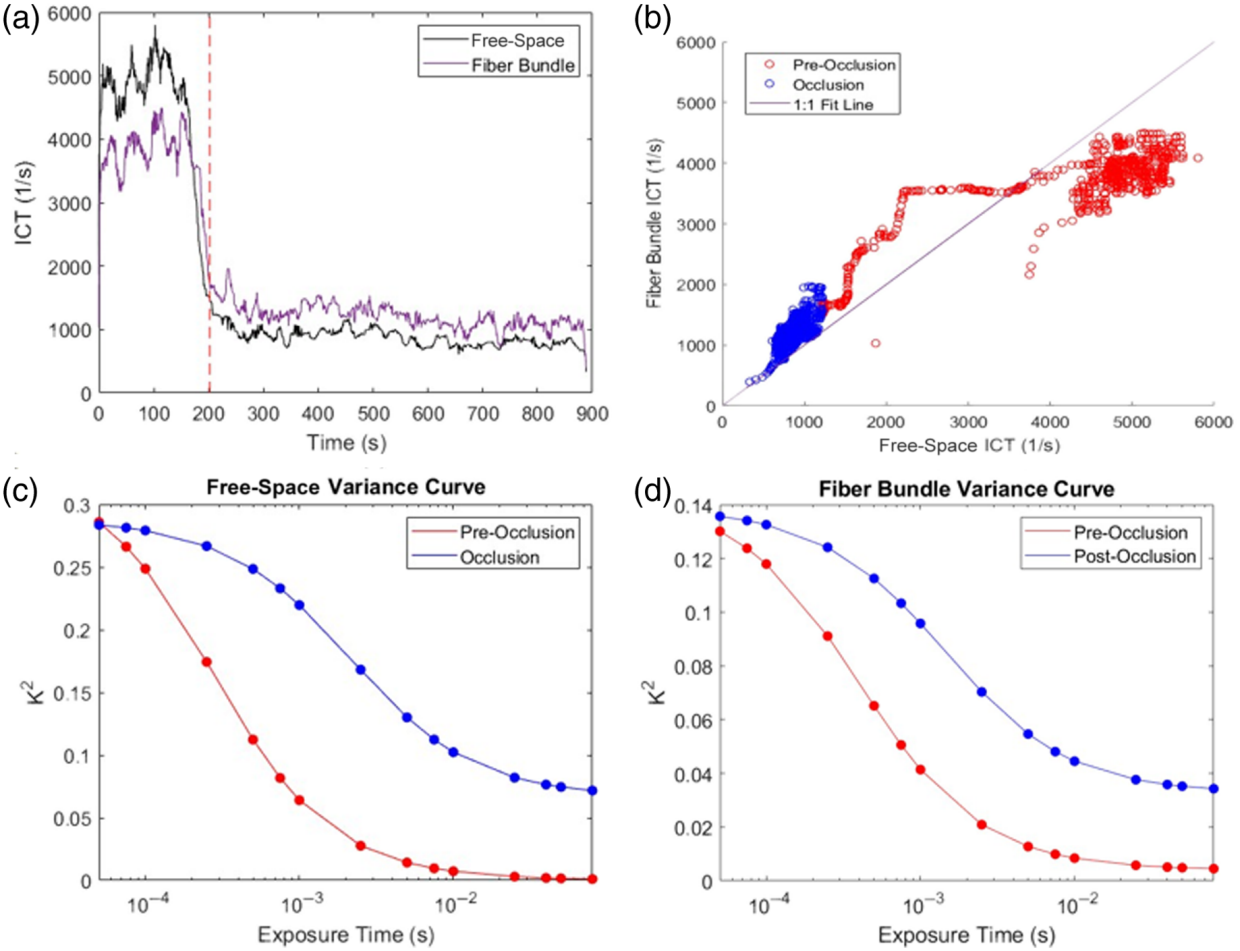
Results of flow rate measurements over the course of stroke induction for vessel 1. (a) ICT plot of flow rate for both the free space geometry and the fiber bundle geometry. The red lines denote when the occlusion fully formed. (b) ICT comparison plot and 1:1 fit line. (c) Speckle variance plot before occlusion and during occlusion for the free space geometry. (d) Speckle variance plot before occlusion and during occlusion for the fiber bundle geometry.

Vessel 1 is the top vessel ROI in Figs. 9(a) and 9(d) that shows a decrease in flow during the stroke with no recurrence of flow afterward. Comparing the time course of ICT values from the free space path and fiber bundle paths using PCC, an $R^{2}$ of 0.953 was found (Fig. 10).

**Fig. 11:**
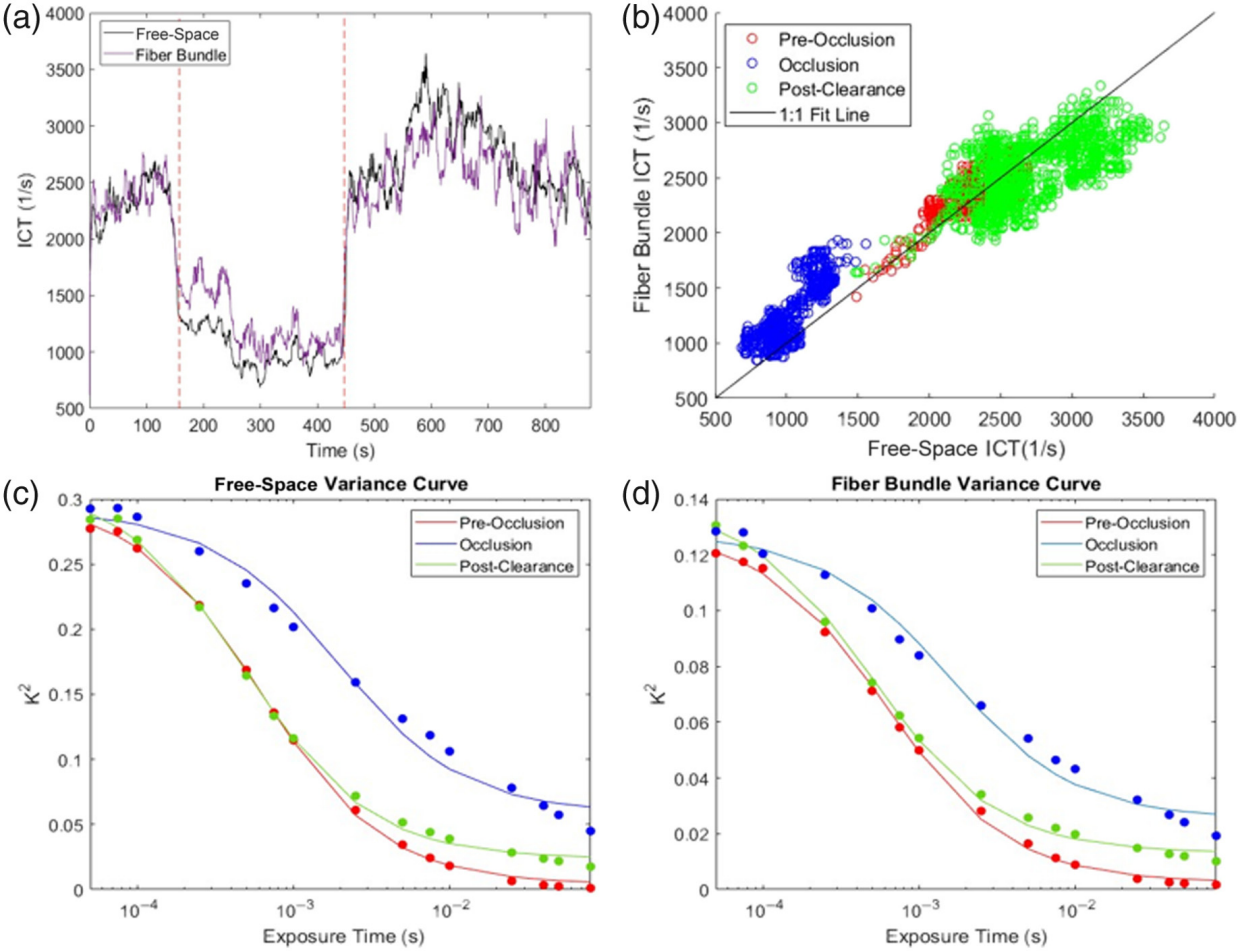
Results of flow rate measurements over the course of stroke induction for vessel 2. (a) ICT plot of flow rate for both the free space geometry and the fiber bundle geometry. The red lines denote when the occlusion was fully formed and blocking blood flow. (b) ICT comparison plot and 1:1 fit line. (c) Speckle variance plot before occlusion, during occlusion, and after clearance of the occlusion for the free space geometry. (d) Speckle variance plot of each flow rate for the fiber bundle geometry.

Vessel 2 is the bottom vessel ROI in Figs. 9(a) and 9(d) that shows a decrease in flow during the stroke, with a return of flow afterwards. Comparing the time course of ICT values from the free space path and fiber bundle paths using PCC, an $R^{2}$ of 0.906 was found (Figs. 11).

## 4 Discussion

### 4.1 Fiber Bundle Speckle Contrast Measurements

Calculating contrast through a fiber bundle via the traditional sliding window for LSCI has been demonstrated to correlate low contrast values with increased flow.^14^^–^^17^^,^^38^ MESI through a fiber bundle has also been done and demonstrates the ability to predict flow with a 22% to 30% error.^15^ The results of this work show that contrast varies significantly more for the traditional sliding window and 1× magnification compared with the nearest fiber approach and 4× magnification. The variation is likely due to pixels sampling different ratios of dead space and fibers, as the organization of the individual fibers does not match a standard 2D camera sensor, resulting in skewed contrast values.

Calculating speckle contrast through a fiber bundle requires special considerations due to the dead space between individual fibers. Regan et al.^16^ studied LSCI through a fiber bundle for examining pulsatile flow in teeth. They calculated contrast spatially and temporarily through the fiber bundle and a free space system. Their findings indicate that temporal contrast values through a fiber bundle decrease with an increase in flow, similar to spatial and temporal contrast responses in the free space system. Spatial contrast, on the other hand, exhibited much larger errors and higher contrast values than any of the other three methods.

Song and Elson^15^ addressed the dead space issue by taking the frequency response of the raw images and removing the dead space via a Butterworth filter. The filter size was dependent on the size of individual speckles. A speckle pattern containing speckles imaged across two or more individual fibers could use a low-pass Butterworth filter, whereas speckle patterns with smaller individual speckles utilized a filter that matched the fiber bundle spatial frequency. They performed a similar masking method to the work described in this paper, but then removed the dead space between fibers through a spatial filter to do sliding window contrast. The method described in this work removes the effects of dead space in the bundle by segmenting individual fibers, then treating individual fibers as samples in the speckle contrast calculations.

A similar approach to the traditional sliding window calculation was utilized by treating each fiber as a sample and calculating contrast using the nearest 36 fibers. This method can be extended to different fiber bundles as long as the proximal end of the bundle is imaged, so individual fibers can be segmented. The number of nearest fibers should be selected based on the organization of the fibers in the bundle and a minimum of 25 to maintain the number of samples used in a 5 × 5 sliding window.

### 4.2 Microfluidic Experiments

Free space MESI has been shown to accurately measure flow in both *in vitro* and *in vivo* settings.^19^^,^^20^^,^^30^ The results of this experiment show that free space MESI and fiber bundle MESI can accurately measure flow in low and high flow rate settings (Figs. 5 and 6). Deviations from the fit line can possibly be explained by unstable flow rates within the pressure pump.^29^ This is further confirmed by the comparison of ICT measurements from both imaging paths as they are highly correlated. Examining the speckle variance curves for either set of flow rates, the speckle variance is lower for the fiber bundle measurements than for the free space measurements. This is possibly due to the averaging within an individual fiber through the segmentation algorithm, reducing standard deviation measurements, in turn reducing speckle contrast. Regardless, through fitting of the MESI equation, similar ICT values were obtained.

### 4.3 *In Vivo* Mouse Experiments

Free space MESI has been shown to accurately measure flow in both *in vitro* and *in vivo* settings.^19^^,^^20^^,^^30^ The results of this experiment show that free space MESI and fiber bundle MESI can accurately measure the flow *in vivo* of the mouse cortical surface. Different regions of the brain, including vessels and parenchyma, show a high correlation between ICT values obtained through both paths. Changes in flow were also shown to have a high correlation between the two paths through the stroke experiment where two vessels were occluded, and any return of flow or lack thereof was observed.

## 5 Conclusion

We have demonstrated a MESI system that utilizes an optical fiber bundle to image speckle patterns. We demonstrate that imaging with a fiber bundle produces similar results to free-space MESI measurements. By treating each fiber as a sample, we can get more accurate results than using a sliding window across the proximal end of the bundle for contrast measurements. For microfluidic measurements at low and high flow rates, we showed that the fiber bundle imaging path had a high correlation between ICT measurements with the free space path. *In vivo* results show that the fiber bundle system can accurately measure flow in the vessel and parenchyma regions of the mouse cortex and can accurately track changes in flow during a stroke.

The fiber bundle imaging utilizes similar hardware and methods as free-space MESI. Using a fiber bundle for imaging, endoscopic systems, or hardware systems where achieving free space imaging is difficult can still utilize MESI for blood flow measurements. The total time of all 15 exposure times is 223 ms and with the readout time is ∼300  ms per MESI sequence. This acquisition time is too long to measure pulsatile flow in mice, considering they have a resting heart rate of 500 to 700 bpm but potentially is short enough for assessing pulsatile flow in humans with a resting heart rate of 60 to 100 bpm. The system used in this paper is not more compact than the free space version due to the use of a beam splitter and the lenses selected for imaging the speckle pattern onto the distal end of the fiber bundle. To achieve a more compact system at the distal end, miniature lenses can be added to the fiber bundle.

## Data Availability

All data in support of the findings of this paper are available within the article.
